# “Estrutura da Celula Nervoza”, by Bruno Lobo and Gaspar Vianna (1908): a pioneering work on Brazilian Neuroscience

**DOI:** 10.1590/0004-282X-ANP-2020-0288

**Published:** 2021-06-14

**Authors:** Bruno Lopes SANTOS-LOBATO, José Eymard Homem PITTELLA

**Affiliations:** 1 Universidade Federal do Pará, Instituto de Ciências da Saúde, Faculdade de Medicina, Belém PA, Brazil. Universidade Federal do Pará Universidade Federal do Pará Instituto de Ciências da Saúde Faculdade de Medicina Belém PA Brazil; 2 Universidade Federal de Minas Gerais, Faculdade de Medicina, Departamento de Anatomia Patológica e Medicina Legal, Belo Horizonte MG, Brazil. Universidade Federal de Minas Gerais Universidade Federal de Minas Gerais Faculdade de Medicina Departamento de Anatomia Patológica e Medicina Legal Belo Horizonte MG Brazil

**Keywords:** Neurosciences, Brazil, History, Neurociências, Brasil, História

## Abstract

Currently, the scientific production in Neuroscience in Brazil is very rich, but, historically, it has been scarce at first. The aim of this study is to present the work “*Estrutura da Celula Nervoza*”, by Bruno Lobo and Gaspar Vianna (1908), as a pioneering work for Brazilian science.

## RESUMO

Atualmente, a Neurociência brasileira demonstra uma pujante produção científica, porém os relatos das primeiras publicações históricas dessa área do conhecimento no Brasil são escassos. O objetivo deste estudo é apresentar a obra “*Estrutura da Celula Nervoza*”, de Bruno Lobo e Gaspar Vianna (1908), como uma obra pioneira para a ciência brasileira.

## INTRODUCTION

**Figure 1.**
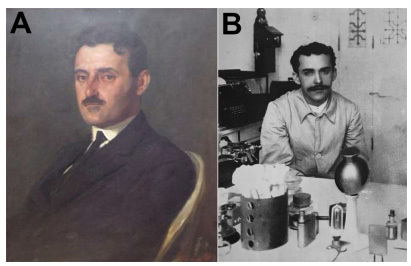
The authors of “*Estrutura da Celula Nervoza*” (1908). (A) Bruno Álvares da Silva Lobo (1884-1945), former director of the Brazilian National Museum, between 1915 and 1923. (B) Gaspar de Oliveira Vianna (1885-1914) at Instituto Osvaldo Cruz.

**Figure 2.**
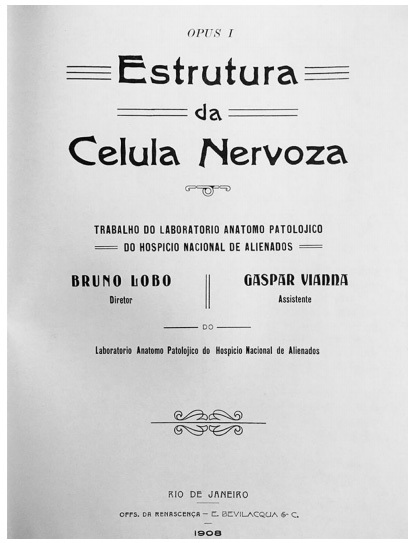
Front page of “*Estrutura da Celula Nervoza*” (1908), reproduced from *Opera Omnia de Gaspar Vianna*, 1962.

In late 19^th^ century and early 20^th^ century, there were advances in histological techniques for the study of the nervous system. In Europe, many works on histology and organization of the nervous system were published, and the monumental work by Santiago Ramon y Cajal, published between 1899 to 1904, is a milestone^1^. In parallel, a 154-page morphological study on the nervous tissue was published in Rio de Janeiro (1908) by the two young physicians born in Pará, Bruno Lobo (1884-1945) (Figure 1A), director of the Anatomopathological Laboratory of *Hospício Nacional de Alienados* (and future director of the Brazilian National Museum between 1915 and 1923), and Gaspar Vianna (1885-1914) (Figure 1B), his laboratory assistant (who would be known afterwards as one of the greatest figures in Medicine and Science in Brazil due to his contributions to the treatment of leishmaniasis and Chagas’ disease)^2^^,^^3^. Both were directly influenced by the works of renowned physicians Juliano Moreira (1873-1933) and Eduardo Chapot Prevost (1864-1907). Their objective was to enlighten about the structure of nervous system elements, and the text was focused in presenting state of the art description of the structure of the nervous system in Portuguese. Here, we aim to discuss the relevance of the work “*Estrutura da Celula Nervoza*” (Structure of the nervous cell) (Figure 2) for Neuroscience in Brazil.

The book was divided in an introduction and five sections: origin and development, generalities, cell body, protoplasmic extensions, and axons and their sheats (“*Orijem e Desenvolvimento*”, “*Generalidades*”, “*Corpo Celular*”, “*Prolongamentos Protoplasmáticos*” and “*Cilindro-eixo e seus envolucros*”). It is richly illustrated, with original microphotographs and schematic images from other authors. Many scientists who published their research on nervous tissue are quoted, but Bruno Lobo and Gaspar Vianna do not list the references cited at the end of the book or the years the scientists cited published their investigations. We describe below the sections of the book.

At the beginning of 20^th^ century, there was a heated debate about the nature of the nervous tissue, whether it would be a single large network (reticular theory) or composed of several cells communicating with each other (neuron doctrine). Iconic authors such as Joseph von Gerlach, Camillo Golgi and Franz Nissl defended the reticular theory, and their positions were criticized by Lobo and Vianna, which were in favor of the neuron doctrine proposed by Cajal^4^. Regarding the possible mechanism of communication between nervous cells, Lobo and Vianna admit the existence of thin anastomoses ("*delgadas anastomozes*") to better establish relations and facilitate their function, which is close to the functional concept of synapse by Sherrington^5^.

## ORIGIN AND DEVELOPMENT

Through the description of neuroblasts in the nervous system of animals, the authors argued that these were precursors of nerve cells in adult animals, reinforcing their support on the neuron doctrine. They discussed the development of the nerve cell processes such as the protoplasmic extensions (dendrites) and the axon (“*cilindro-eixo*”). They also commented on the origin of the distal end of the axon: if it originated from a local neuroblast or from an emigrated neuroblast and differentiated in a Schwann cell.

## GENERALITIES

In this session, the authors commented on the morphological classification of the nerve cells as proposed by Cajal. They pointed out the great variability of dimensions and shapes of the cell body and its extensions in comparison to other cell types of the organism.

## CELL BODY

This is the main session of the book. Even being a morphological study, Lobo and Vianna tried to suggest the function of some organelles. For example, the authors speculated that the “*microzoma*” (actually, mitochondria, not rough endoplasmic reticulum) of nerve cells would have no important function in cell biology, with a rather secondary function with minor importance (or, in their words, “*de função secundaria e de somenos importancia*”). They also address the “*granulações de Nissl*” (known as Nissl substance and corresponding to the rough endoplasmic reticulum at the electron microscopy) as a differentiation of the protoplasm, with functions related to the functional and nutritious energy of the element (“*enerjia funcional e nutritiva do elemento*”). Currently, we know that the rough endoplasmic reticulum is essential for protein synthesis. The authors correctly described the presence of a natural brown pigmentation in some nuclei of the brainstem, including the substantia nigra, and correctly suggested it was melanin, which gradually increases until the individual’s complete development. Also, they commented on the recent and exciting discovery of neurofibrils, whose proposed function was to transmit the nervous influx (“*transmitir o influxo nervozo*”), and how pathological states (such as intoxications and infections) could alter the structure of neurofibrils. It is known, nowadays, that the neurofibrils are actually aggregates of microtubules and neurofilaments responsible for axonal transport.

## PROTOPLASMIC EXTENSIONS

Dendrites were confirmed as extensions of the protoplasm by the authors. They commented on small extensions originated from dendrites, called by various names at the time, including the term “spine” ("*espinho*"), without a known function.

## AXONS AND THEIR SHEATHS

Lobo and Vianna reviewed the current conceptions about the axon and the double lipid-protein nature of myelin protein, including a specialized structure called “*estrangulamento anular de Ranvier*” (currently known as node of Ranvier), and suggested a physical contact between myelin borders. About Schwann cells, the authors agreed with Ranvier's opinion that the Schwann cell produces myelin, disagreeing with Kölliker and Cajal, who stated that it originated from the axon. They also argued that, in the central nervous system, due to the absence of the Schwann cell, myelin may have a different origin from the myelinated fiber of the peripheral nervous system. At that time, Cajal admitted the existence of Schwann cells in the central nervous system, claiming that they were very delicate, requiring the use of elective staining methods and high magnification to identify them under the microscope. It was only later on, between 1919 and 1921, that the Spaniard neuroscientist Pío del Río Hortega (1882-1945) discovered the oligodendrocyte, responsible for the synthesis of myelin in the central nervous system.

Unfortunately, the pioneering initiative of Bruno Lobo and Gaspar Vianna to publish a work with the most modern discoveries about the morphology of the nervous system in Portuguese and in books or booklets with low circulation went unnoticed by the national and international scientific community. Furthermore, they were young researchers (both were younger than 30 years old) from a country without tradition in neurological research. At the best of our knowledge, there were no similar works in Brazil before 1908. After 1908, “*Estrutura da Celula Nervoza*” was inserted in a collection published in 1962, comprising all scientific works by Vianna’s, being the main source for consulting the original full document nowadays^6^. We hope the work and life of Lobo and Vianna can be an inspiration for modern generations of medical scientists.

## References

[1] 1. Cajal RS. Textura del sistema nervioso del hombre y los vertebrados. Madrid: Moya; 1899-1904.

[2] 2. Fraiha Neto H. O centenário de nascimento de Gaspar Vianna. Rev Soc Bras Med Trop. 1986 Apr-Jun;19(2):111-33. https://doi.org/10.1590/S0037-86821986000200013 10.1590/s0037-86821986000200013 3324214

[3] 3. Pittella JEH. The remarkable pioneering contribution of Gaspar Vianna to the study of the neuropathology of Chagas disease. Arq Neuro-Psiquiatr. 2018 Dec;76(12):853-856. https://doi.org/10.1590/0004-282x20180137 10.1590/0004-282X20180137 30698210

[4] 4. Guillery RW. Observations of synaptic structures: origins of the neuron doctrine and its current status. Philos Trans R Soc Lond B Biol Sci. 2005 Jun;360(1458):1281-307. https://doi.org/10.1098/rstb.2003.1459 10.1098/rstb.2003.1459 PMC1569502 16147523

[5] 5. Shepherd GM, Erulkar SD. Centenary of the synapse: from Sherrington to the molecular biology of the synapse and beyond. Trends Neurosci. 1997 Sep;20(9):385-92. https://doi.org/10.1016/s0166-2236(97)01059-x 10.1016/s0166-2236(97)01059-x 9292963

[6] 6. Falcão EC. Opera Omnia de Gaspar Vianna. São Paulo: Empresa Gráfica da Revista dos Tribunais; 1962.

